# Effect of Slat and Gap Width of Slatted Concrete Flooring on Sow Gait Using Kinematics Analysis

**DOI:** 10.3390/ani9050206

**Published:** 2019-04-30

**Authors:** Nicolas Devillers, Emmanuel Janvier, Farhoud Delijani, Steve Méthot, Kristopher J. Dick, Qiang Zhang, Laurie Connor

**Affiliations:** 1 Agriculture and Agri-Food Canada, Sherbrooke Research and Development Centre, 2000 College Street, Sherbrooke, QC J1M 0C8, Canada; e.janvier@groupe-esa.net (E.J.); steve.methot@canada.ca (S.M.); 2 Department of Biosystems Engineering, University of Manitoba, Winnipeg, MB R3T 5V6, Canada; farhoud.delijani@umanitoba.ca (F.D.); Kristopher.Dick@umanitoba.ca (K.J.D.); Qiang.Zhang@umanitoba.ca (Q.Z.); 3 Department of Animal Science, Faculty of Agricultural and Food Sciences, University of Manitoba, Winnipeg, MB R3T 2N2, Canada; Laurie.Connor@umanitoba.ca

**Keywords:** kinematics, lameness, lattice design, slatted concrete floor, pig

## Abstract

**Simple Summary:**

The housing of gestating sows is currently moving from individual stalls to group housing in most Western countries. If the design of slatted concrete floors was well studied for smaller animals such as finishing pigs, less information is available for sows. Slatted concrete floors can have negative effects on sow health, especially in connection with lameness. The aim of this study was to determine the optimal slat and gap widths of slatted concrete floors for the ease of movement of sows. After testing several combinations of slat and gap widths and quantifying the gait of walking sows using kinematics analysis, we determined that the usual 25-mm gap/125-mm slat commonly used in North America may not be optimal to facilitate the ease of movement of sows. In particular, sows’ gait was less affected by smaller gap widths. Further research is now necessary to assess the impact of floor design on behavior, lameness occurrence, performances and longevity of sows.

**Abstract:**

The housing of gestating sows in groups requires sound information about the adapted design of the pen floor. Slatted concrete floors are commonly used for effective drainage of manure but can cause feet injuries and lameness. In the present study, kinematics were used to characterize the gait of 12 gilts and 12 lame sows walking in a corridor on slatted concrete floors with different combinations of slat (85, 105 or 125 mm) and gap (19, 22 or 25 mm) widths. The nine experimental floors were tested with slats in the perpendicular and parallel orientation to the direction of animal walk, according to a duplicated lattice design. Gait parameters were quantified using spatial, temporal and angular kinematics for front and rear limbs. Some parameters were significantly affected by the treatments (*p* < 0.05), but the effects differed between gilts and lame sows and between slat orientations. Gap width had a significant effect on parameters such as back angle, stride length, foot height, and carpal and tarsal joint angle amplitudes. Slat width significantly affected parameters such as foot height, and carpal and tarsal joint angle amplitudes. Comparisons of the different combinations of slat and gap widths revealed that slats with a width of 105–125 mm and gap width of 19–22 mm had the least effect on the gait characteristics of the gilts and sows.

## 1. Introduction

Following the ban on group housing for gestating sows in the European Union and other countries, Canadian producers have engaged in a move from individual stalls to group housing systems for gestating sows by 2024 [1]. Since most of the existing sow barns are designed for a slurry manure management system, slatted concrete floors are commonly used in gestation barns for manure drainage. However, sows that are group-housed on slatted floors are more prone to foot lesions than stall-housed sows on similar floors [2,3]. Therefore, the best design of a slatted floor for free moving sows in a group-housed system is most likely not the same as that for constrained stall-housed sows. Slatted floors have been shown to increase the occurrence of lameness and claw lesions in group-housed sows compared to solid or bedded floors [4,5,6,7]. Therefore, there is a need to optimize the design of slatted floors to reduce the negative impact on sow feet and leg health.

Slat design has been extensively studied for manure drainage or ammonia emission in piggeries [8,9,10,11], and slat and gap widths are often based on the efficiency of manure drainage. The overall permeability of the floor, which takes both gap width and percentage of slatted area into account, should be at least 12% [9]. For example, in North-American commercial piggeries, slats usually measure five inches (125 mm) and gaps are one inch (25 mm) in width, with a permeability of about 17% for a fully slatted floor.

Few studies looked at the impact of a slatted concrete floor design on pig performance and welfare. Some studies compared fully versus partially slatted floors for fattening pigs [9,12,13,14]. Rähse and Hoy [15] found a tendency for more severe claw lesions in fattening pigs kept on slatted floors with a gap wider than 20 mm. Whereas Falke, et al. [16] found no effect of the slat width on leg and foot lesions between 153- and 190-mm slats (both with gaps of 15 mm). For gestating sows, specific studies are even more scarce, while recommendations for gap or slat widths vary widely between 17 and 30 mm for gaps and 80 and 200 mm for slats [14].

Specifications of slatted concrete floor are regulated in the European Union and a maximum gap width of 20 mm and a minimum slat width of 80 mm are required for pregnant gilts and sows [17]. The Canadian code of practice does not provide any specific recommendation but asks to ensure appropriate gap widths for the sizes of pigs and slat widths that maximize the contact area with the foot sole [1]. Indeed, recommendations on slat and gap widths vary with the size of the animal [17], most likely because the size of the foot is proportional to the body weight and the void percentage of the slatted floor should be a function of live weight [13,18]. Since the body weight of pregnant gilts and sows varies greatly from less than 150 kg to more than 250 kg [19], slats designed for multiparous sows may not necessarily accommodate gilt needs. Indeed, the Scientific Panel on Animal Health and Welfare [20] recommended that “the width of a gap should not exceed half the width of the contact area between the foot and the floor and the solid area between the gaps should be sufficient to support the foot.” Anatomical measurements show that claw widths average 25–30 mm in adult sows, while sole or toe lengths measure near 45–51 mm [13,21]. This would speak in favor of Tubbs’ [22] recommendation of a gap of 13–16 mm rather than 19–25 mm to minimize injuries to toes and dew claws. On the other hand, slat width therefore should measure at least the sole length or twice the claw width, which would be in average more than 60 mm.

The objectives of the present study were to characterize the gait of gilts, which have smaller feet, and lame sows, which have more difficulty to walk, on slatted floors with different combinations of slat and gap widths in order to determine the optimal floor design for group-housed sows. The hypothesis was that an adequate slatted floor must not impair the gait of the pigs compared to a solid concrete floor. In essence, smaller gaps and larger widths of slats may result in better locomotion as this kind of configuration is closer to a solid floor.

## 2. Materials and Methods 

Animals were cared for according to a recommended code of practice [1] and the experimental protocol F14-031 was approved by the institutional animal care committee of the University of Manitoba (Winnipeg, MB, Canada), in accordance with the Canadian Council on Animal Care guidelines [23].

### 2.1. Animals

Twenty-four Yorkshire × Landrace sows and gilts were selected according to their size and their severity of lameness and categorized in two groups. The first group, called “Sound Gilts” (SGs), included young and small animals of parity 0 or 1 (mean ± SD: 0.5 ± 0.5) with a visual gait score of 0 at the beginning of the experiment. The second group, called “Lame Sows” (LSs), included primiparous and multiparous sows (mean parity ± SD: 2.5 ± 1.4), which were usually larger, taller and heavier than SGs and had a visual gait score between 1 and 3. The visual gait scoring method was adapted from Main, et al. [24] as proposed by Conte, et al. [25], with which lameness is assessed on a scale from 0 to 4 (0: normal gait; 1: abnormal stride length is detected; no obvious lameness; 2: stride is shortened and lameness is detected; 3: sow does not place affected limb on the floor; 4: non-ambulatory sow). In total, 12 SGs and 12 LSs were selected to conduct the experiment. Table 1 summarizes the characteristics of the animals. Sound gilts and lame sows were housed within groups of 20–24 individuals on a partial slatted floor (125-mm slats, 25-mm gaps) with an automatic feeder.

### 2.2. Treatments

**Figure 1:**
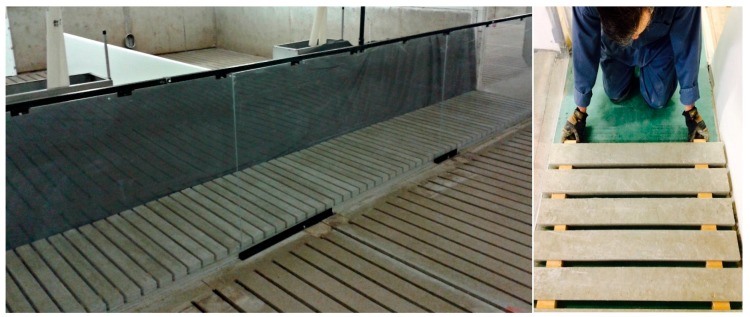
Set-up of corridor for kinematics measurement.

Nine experimental floors corresponding to the combinations of three slat widths (85, 105 and 125 mm) and three gap widths (19, 22 and 25 mm) were tested in two orientations (Figure 1). Slats were either parallel or perpendicular to the direction of the walk of the animal along the test corridor (7 m in length and 1 m in width). Additionally, control floors, which simulated the solid concrete floor, were installed with concrete slats of 125 mm placed side by side without any gaps between slats and tested in both orientations (parallel and perpendicular to the direction of animal walk).

The concrete slats were manufactured by a commercial concrete company (Lafarge, Winnipeg, MB, Canada), with the formworks designed by the research team. Individual concrete slats were cast in three widths of 85, 105 and 125 mm, with a depth of 50 mm and length of 915 mm. No conventional reinforcement such as steel or GFRP (Glass Fiber Reinforced Polymer) bars was used in the slats. Fiber-reinforced, 25-MPa concrete made by Lafarge was used to cast the slats. All cast slats were cured for 7 days at the Lafarge plant before transportation to the hog barn. Slats were stored in the barn for another three weeks before installation for tests. 

The corridor pathway floor was fixed and leveled first to make sure it was perfectly flat. Test slats were laid down by hand over the leveled pathway. Three different sizes of wooden spacers (19, 22 and 25 mm) were used to create the desired gaps in between the slats (Figure 1). Slats were removed after each test and a new orientation was laid down for the next test.

### 2.3. Experimental Design

Experimental floors were compared according to a lattice design (balanced incomplete block design) repeated for each type of animal (SG or LS) and each orientation of floor (parallel or perpendicular). The lattice design allowed for testing the nine floors with 12 animals where each animal was assigned to three different combinations of the slat and gap treatments so that each of the nine combinations was tested with four different animals (Table 2). Each animal is considered as a block. Each lattice design was carried out over a week for a total of 4 weeks with one orientation and six SGs and six LSs tested in each week (days 1, 3 and 5). Animals were also walked on control floors on day 2 of each week and were visually scored for lameness on day 4 of each week. Therefore, each LS and SG walked twice on a control floor and was scored twice for lameness.

### 2.4. Kinematics Measurements

**Figure 2:**
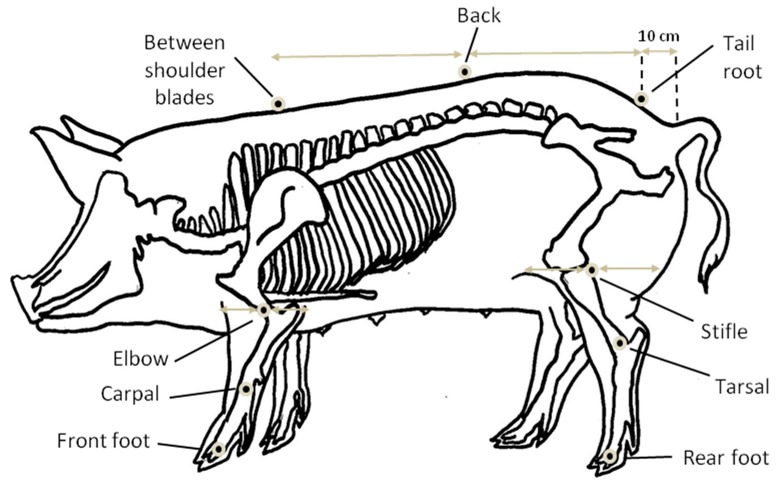
Positions of the nine reflective markers on the sow (three on each leg and three on the back) for the kinematics (feet markers are placed just below the coronary band; carpal and tarsal markers are placed on the radio-carpal and tibio-tarsal joints, respectively; elbow marker is in the middle of the fore leg sides at the elbow joint level; stifle marker is in the middle of the hind leg sides at the stifle joint level; and the back marker is exactly in between the shoulder blades and tail root markers [25]).

Each animal was video recorded while walking along the corridor (3.9 m in length) delimited by a black plastic panel and a clear acrylic glass panel on each side and where the experimental floor was installed (Figure 1). Nine reflective markers (plastic balls of 12.7 mm with reflective tape (B & L Engineering, Santa Ana, CA, USA) were placed in standardized spots of the sow’s body: three on each leg from one side and three on the back according to the same method described by Conte et al. [25] (Figure 2). Sows were video-recorded either on the left side or the right side, but always on the same side for the three treatments per sow. Sows were trained to walk in the corridor following a stock person until they were used to walking at a steady pace. The stock person prevented the sow from running and could use a bucket with feed to entice her to walk. In total, 12 animals were recorded on the left side and 12 on the right side using a DFK22AUC03 camera (The Imaging Source Europe GmbH, Bremen, Germany) with lens (Pentax CCTV C418DX, 4.8 mm, 1:1.8; Pentax Ricoh Imaging Americas Corporation, Denver, CO, USA) and the IC Imaging Capture 2.2 software (The Imaging Source LLC, Charlotte, NC, USA). Gait characteristics were analyzed using MoviAS pro software (NAC Image technology, Simi Valley, CA, USA). The parameters measured were selected according to Grégoire et al. [26] and Conte et al. [25]: walking speed, stride length, swing time (when the foot was in movement, off the floor), stance time (when the foot was in contact with the floor), foot height (the foot’s maximum height during the swing), and angle mean and amplitude for the carpal (fore leg) and tarsal (hind leg) joints during the swing and stance periods. Data from 3 to 4 steps were averaged for each parameter.

### 2.5. Statistical Analyses

Statistical analyses were carried out using SAS (SAS Inst. Inc., Cary, NC, USA) with the gilt (or sow) as the experimental unit. For control floors, half of the animals were tested on floors with parallel orientation first, and the second half on floors with perpendicular orientation first. Impact of the orientation of the slats was first tested according to a crossover design using the orientation, the week and their interaction as fixed factors and revealed no significant effect of the orientation. Therefore, repeated measures analyses were performed using the MIXED procedure with the type of animal (SG vs. LS), the week and their interaction as fixed factors and the animal as random factor. Results are expressed as least square means ± SEM.

Each parameter was analyzed separately for each orientation of the slats (parallel or perpendicular), each type of animal (SG or LS) and each type of limb (front or rear). Potential covariates were tested for linear relationship with response variables and absence of interaction with treatments. These covariates were the body height (hmax: maximal distance between the front foot and the shoulder blades markers) and the body length (lmax: maximal distance between shoulder blade and tail root markers) of the sow when walking. Full models of analyses of covariance (ANCOVA) using the MIXED procedure were carried out including the interactions between treatment and the covariates and the single effect of the covariates. Significant covariates were kept in the reduced model where the treatment effect was separated according to its factorial structure with slat width, gap width and their interaction as fixed effects. Random effects were the replication of the lattice design and the animal (block within replication). When either of the factors (slat or gap width) was significant, pairwise comparisons between the three levels of factors were performed using a Tukey adjustment. Results were expressed as least square means ± SEM for the four animals per treatment combination. When covariates were significant for some parameters, comparisons were made at the mean value of the covariate and the least square means and SEM were corrected accordingly.

## 3. Results

### 3.1. Gait Characteristics of Sound Gilts and Lame Sows on Control Floors

Table 3 presents the results for the analysis on control floors (solid). Only a few gait parameters were different between the two types of animal. Sound gilts had a wider angle of the back (SG: 172° vs. LS: 166°, F_1,22_ = 8.1, *p =* 0.010) and a shorter stance time for the front limbs (SG: 598 ms vs. LS: 692 ms, F_1,22_ = 6.4, *p =* 0.019) and the rear limbs (SG: 590 ms vs. LS: 696 ms, F_1,22_ = 8.4, *p =* 0.008) than lame sows. Some parameters were different between the two types of animal for one of the two weeks only. Sound gilts had a wider tarsal angle during the swing phase on week 1 (F_1,22_ = 5.7, *p =* 0.026) and a higher amplitude of variation of the tarsal joint angle during the stance phase on week 2 (F_1,22_ = 4.3, *p =* 0.051) compared to lame sows.

### 3.2. Effects of Slat and Gap Width on Gait Characteristics of the Animals

Table 4 and Table 5 show the results from the analyses of kinematic parameters by orientation of the slats (parallel or perpendicular) and type of limbs (front or rear) for SGs and LSs separately. Parameters for which no results are reported were not affected by treatments (*p* ≥ 0.10).

#### 3.2.1. Slat and Gap Width Effects on Sound Gilts

Results from the present study show that SG gait was significantly affected by slat width for the front foot height in both parallel (F_2,15_ = 3.8, *p =* 0.047) and perpendicular (F_2,16_ = 5.7, *p =* 0.014) orientations, and for the carpal joint angle amplitude during stance (F_2,16_ = 3.7, *p =* 0.048) when slats were in parallel orientation. Front foot height was higher for 105-mm slats compared to 125-mm slats when parallel (5.4 vs. 4.4 cm respectively, t_15_ = 2.62, *p =* 0.048) and higher for 105-mm slats compared to 85- and 125-mm slats when perpendicular (5.7 vs. 4.7 (t_16_ = −2.97, *p =* 0.023) and 4.7 cm (t_16_ = 2.85, *p =* 0.029), respectively). Carpal angle amplitude was higher during stance for 85-mm slats compared to 105-mm slats in the parallel orientation (13.8° vs. 12.1° respectively, t_16_ = 2.62, *p =* 0.047).

For SGs, the gaps width had a significant effect on their back angle (F_2,16_ = 4.0, *p =* 0.040) when slats were in the perpendicular orientation and on the carpal joint angle amplitude during stance (F_2,16_ = 4.4, *p =* 0.031) when slats were in the parallel orientation. The back angle was wider for 19-mm gaps compared to 25-mm gaps in the perpendicular orientation (172.1° vs. 169.7° respectively, t_16_ = 2.67, *p =* 0.042). The carpal angle amplitude was higher during stance for 22-mm gaps compared to 19- and 25-mm gaps in the parallel orientation (14.2° vs. 12.6° (t_16_ = −2.51, *p =* 0.057) and 12.5° (t_16_ = 2.59, *p =* 0.049), respectively). Finally, walking speed tended (F_2,16_ = 3.19, *p =* 0.068) to decrease with increasing gap width.

Two parameters were significantly affected by the interaction between slat and gap widths on parallel-oriented slats, i.e., the stride length (F_4,6_ = 8.5, *p =* 0.012) and foot height (F_4,7_ = 6.5, *p =* 0.017) of rear limbs for SGs. However, for these two parameters, there was also a significant interaction between the treatments and the covariate (body length, F_8,6_ = 6.23, *p =* 0.019 and F_8,7_ = 3.85, *p =* 0.046, respectively). In this case, the least square means are preferably presented for three values of the covariate, which would result in 3 values × 3 slat widths × 3 gap widths, i.e., 27 least square means for each parameter. Therefore, in order to simplify the results and facilitate the interpretation of the results, the least square means given in Table 4 are for the mean value of the covariate and slice effects for separate gap or slat widths were calculated. For the stride length of rear limbs for SGs walking on parallel slats, there was a significant gap width effect for the 105-mm slat only (F_2,6_ = 5.22, *p =* 0.049), stride being longer when gap width decreased. For the foot height of rear limbs for SGs walking on parallel slats, there was a slat width effect for every gap width (19 mm: F_2,7_ = 4.66, *p =* 0.052; 22 mm: F_2,7_ = 11.63, *p =* 0.006; 25 mm: F_2,7_ = 7.66, *p =* 0.017), but the effect was inconsistent depending on the gap width. Overall, with the exception of the front limb foot height and the mean back angle, most of the significant effects were seen when slats were parallel.

#### 3.2.2. Slat and Gap Width Effects on Lame Sows

Lame sows had their gait significantly affected only when the slats were in perpendicular orientation (Table 5) with a slat width effect for the carpal (F_2,16_ = 4.6, *p =* 0.027) and tarsal (F_2,16_ = 5.5, *p =* 0.015) joint angle amplitudes during swing. Both the carpal angle amplitude and the tarsal angle amplitude were higher in LSs during swing for 125-mm slats compared to 105-mm slats in the perpendicular orientation (carpal: 56.3° vs. 49.0° respectively, t_16_ = −2.89, *p =* 0.027; tarsal: 34.0° vs. 29.2° respectively, t_16_ = −3.06, *p =* 0.019).

Moreover, the gap width affected the front foot height (F_2,16_ = 5.3, *p =* 0.018) and the tarsal joint angle amplitude during stance (F_2,16_ = 4.9, *p =* 0.022). The front foot height was higher for 25-mm gaps compared to 19-mm gaps in the perpendicular orientation (5.3 vs. 4.3 cm, t_16_ = −3.18, *p =* 0.015, respectively), whereas the tarsal angle amplitude was higher during stance for 22-mm gaps compared to 19-mm gaps in perpendicular orientation (12.8° vs. 10.7°, t_16_ = −2.57, *p =* 0.051, respectively). Overall, contrary to SGs, for LSs, all the significant effects on gait characteristics were seen when slats were perpendicular.

## 4. Discussion

### 4.1. Gait Characteristics of Sound Gilts and Lame Sows

Although the gaits of SGs and LSs were different in only a few parameters, similar differences between lame and sound sows were already observed in studies using the same equipment [25,26], with sound sows presenting a shorter stance time or a wider tarsal angle. An arched back was also more pronounced in lame sows [26]. On the other hand, several parameters were affected by the week in both types of pig. This could compromise the interpretation of the eventual effects of the different slatted floors tested since the difference might be attributable to the evolution of lameness of the animal or to other unknown parameters. Indeed, the between-days variations (%CV) of some kinematics parameters for the same sow can exceed 10% or even 15% [26].

The present study used gilts and small sows, expecting that the anatomy and conformation of these animals could make them more prone to lameness on slatted flooring. Indeed, gilts have smaller feet that may get more easily trapped in wide gaps and be associated with the higher culling rate for lameness observed in first parity sows [27]. On the other hand, “lame sows” were sows that show a modified gait according to several indicators visually observed such as reduced stride length or avoidance to bear weight on a foot. Generally, these sows do not show an easy, regular and symmetrical gait. Consequently, lameness usually translates into modifications of several gait parameters recorded by kinematics such as a shorter stride length, a longer stance time, a slower walking speed, or a lower joint flexion [25,26,28]. Lameness can be due to various pathologies such as claw lesions, osteochondrosis or arthritis that may affect one or several limbs and that are generally painful [29]. Therefore, lame sows are a particularly relevant population of animals that may be more sensitive to variations in floor characteristics. Our results showed that 15 gait parameters tended to be or were significantly affected by the slat and gap width treatments in SGs, while only eight parameters were affected in LSs, and six of these on the perpendicular-oriented slats. Therefore, gilts may be more sensitive to slat and gap widths, most likely due to their anatomy.

### 4.2. Effects of Slats and Gaps Width on Gait Characteristics of the Animals

Of the more than 72 parameters analyzed, only 10 parameters were significantly affected by treatments (*p* < 0.05) and another 13 parameters showed tendencies (*p* < 0.10). The parameters that were significantly affected were related to the back angle, foot height, stride length, and carpal and tarsal joint angle amplitudes. No significant effects were seen on temporal parameters such as swing time or stance time which were previously reported to be affected by lameness severity [26,28,30] or floor conditions [31,32].

In previous research, the stride length was found as a good indicator of natural or induced lameness [26,30] or gait adaptation on a slippery floor [32,33], with a reduction of the stride length when compared to control animals or conditions. A narrower back angle, often visually observed as an arched back, was also reported as a good indicator of lameness in sows [26] and cows [34]. In the present study, these two parameters were significantly affected in gilts only when walking on slatted floors with 19-mm gaps, with longer strides (on the 105-mm slats only) and a wider back angle when walking on the smaller gap compared to 25-mm gaps.

The effects on some parameters are more difficult to interpret due to less information being available in the literature. For example, the foot height was not shown to be modified by factors such as floor slipperiness [33], or lameness [25,26,28] in sows. In cows, the hoof height was affected by the floor surface with a higher foot elevation on rubber floor than concrete floor for both cows with and without sole ulcers [35]. This higher elevation of the stride, along with several other parameters, was interpreted as better comfort and easiness at walking. However, another study found no effect of flooring type (concrete, rubber or resin aggregate) on the foot height [36]. On the contrary, in horses, induced lameness resulted in a higher elevation of the hoof when trotting [37], but this effect was less pronounced when walking [38]. In the present study, the foot elevation was affected differently between gilts and sows depending on the slat orientation. In SGs, both front and rear feet elevations on parallel-oriented slats were similarly affected with a higher elevation when walking on the 105-mm slats, compared to 125-mm slats. On perpendicular-oriented slats, only the front feet height was more elevated on the 105-mm slats. In LSs, the front feet height increased when walking on 25-mm gaps compared to 19-mm gaps of the perpendicular-oriented slats. For sows and gilts on the control floors, the feet elevation was between 4 and 5 cm, thus elevations higher than 5 cm (up to 6 cm) observed in animals walking on 105-mm slats or 25-mm gaps could be interpreted as a deviation from the normal gait. However, other parameters must be considered before a full interpretation can be made.

As for angular parameters, only the amplitudes of carpal and tarsal joint angle were significantly affected by treatments. There is virtually no information available on the biomechanics of walking sows, while information is available for horses [39]. Carpal and tarsal joint movements involve flexion during the swing phase and extension during the stance phase. Consequently, the angle should reach its minimal value during flexion and maximum during extension. On the other hand, the angle variation and amplitude should be maximal during the swing movement when the limb goes from stretched position to bent and back to stretched position, while the limb stays quite stretched most of the stance phase, especially the front limb [40,41]. The values reported in Table 3 for joint angle amplitudes are in accordance with these theoretical principles. In the present study, the locations of reflective markers to assess carpal and tarsal joint variations (front feet/carpal/elbow and rear feet/tarsal/stifle) most likely measured a composite movement of both fetlock and carpal/tarsal joints (see figure 2). Previous research reported that sows lame on a front leg had a lower carpal joint flexion (which would translate in a lower joint angle amplitude during the swing phase), which was indicative of stiffness [28]. Accordingly, Thorup, et al. [42] showed that pigs walking on slippery floors have a lower peak joint moment for forelimbs (equivalent to a lower joint amplitude), indicative of gait adaptation. In the present study, joint movements were differently affected between LSs and SGs and between swing and stance phases of the stride. In SGs, only the carpal joint angle amplitude was modified during the stance phase on parallel slats. As reported by Thorup, Laursen and Jensen [42], gait adaptation to floor conditions was more visible on front limb joints which carry more weight while no change was seen on hind limb joints. Higher carpal joint angle amplitudes during the stance phase could be interpreted as better stretching movement of the limb, and therefore a better ease of movement which was seen for 85-mm slats and for slats with 22-mm gaps in parallel orientation. In LSs, treatments affected both carpal and tarsal joint amplitudes during swing phase with higher flexion on perpendicular-oriented 125-mm slats, indicative of a better ease of movement. Finally, as for gilts, the higher tarsal joint angle amplitude during the stance phase in LSs could be interpreted as a better ease of movement when walking on 22-mm gaps of perpendicular-oriented slats.

Considering all these results together, there was no consistent and very clear effect of slat or gap width on most of the gait parameters studied. Moreover, depending on the parameters considered, effects of gap or slat width differ between SGs and LSs, and between orientation of the slats. Interpretation is therefore difficult, all the more that the information available from previous studies in pigs is scarce. Looking at the values when walking on control floors can further help to look at gait modification in terms of least deviation from the normal gait of the animals. Of the angular parameters significantly affected by slat and gap width, gait characteristics deviated least from solid floor measurements mainly for 105-mm slats for both SGs and LSs. Comparing the results of the current study with and other species, the effects seen tend to show a better ease of walking for the smaller gaps (19 and 22 mm), and the larger slats (105 and 125 mm). Overall, this observation is in accordance with the recommendation of the European Union for a maximum gap width of 20 mm but supports use of a wider slat than the minimum slat width of 80 mm [17]. Further, it suggests that the commonly used slat of 125 mm in North America could be reduced, with a 19-mm gap being particularly suitable for breeding gilts.

### 4.3. Impact of other Factors

#### 4.3.1. Differences between Front and Rear Limbs

More parameters were affected by floor treatments for front limbs (n = 13) than in rear limbs (n = 7) for both slat orientations. Applegate, et al. [43] reported that front limbs were more susceptible to being affected by the floor surface than the rear limbs. Moreover, sows bear approximately 60% of their body weight on the front limbs as their center of gravity is closer to the front limbs [25,44]. Thus, these limbs are more likely to be affected and their gait characteristics to be impaired when the floor is inadequate.

#### 4.3.2. Differences between Parallel and Perpendicular Orientations of the Slats

Although the experimental design did not allow to test the statistical effect of the orientation of the slats, it is worth noting that all the parameters that were significantly affected by slat or gap width were on perpendicularly oriented slats in LSs, while most of the significant effects of slat or gap width in SGs were seen in parallelly oriented slats. On the perpendicularly oriented slats, four parameters were affected in common for SGs and LSs, namely, front foot height, swing carpal angle, stance carpal angle and stance tarsal angle amplitude. It could be hypothesized that gilts and sows could visually perceive the floor design of different slat orientations (perpendicular versus parallel), and thus the slat orientation could influence their willingness to walk. However, previous research showed that the orientation of shadow stripes or black and white lines on the floor had no effect on piglet willingness to move forward [45]. Thus, parallel and perpendicular slots could be felt differently by the animal while walking, and LSs seemed to better feel a difference between slat and gap width when perpendicularly oriented. This would be in accordance with the recommendation to position slats perpendicularly to the walking direction in corridors and alleys [46].

## 5. Conclusions

Based on the data from the kinematics study of the gait characteristics of sound gilts and lame sows walking on various slatted concrete floors, it was found that some gait parameters were significantly affected by the combinations of slat and gap width. Most of the effects were found on the front limbs which bear 60% of the body weight. Sound gilts seemed more sensitive to the variation in the configuration of the slatted floors, showing more modifications of their gaits than lame sows. Comparisons of the different combinations of slat and gap widths revealed that larger slats with a width of 105–125 mm and smaller gap widths of 19–22 mm had the least effect on the gait characteristics of the gilts and sows. Finally, the slat orientation altered more gait parameters in gilts than in sows.

This study was the first step in evaluating the impact of slat and gap widths on the locomotion of sows. However, in order to fully validate slatted floor configurations for group-housed gestating sows, more research should be conducted on the impact of floor design on behavior, lameness occurrence, performances and longevity of sows, as well as pen cleanliness and manure drainage, both in experimental and commercial settings.

## Figures and Tables

**Table 1:** Characteristics of the groups of animals tested: sound gilts (n = 12) and lame sows (n = 12).

Characteristic	Modalities	Sound Gilts	Lame Sows
Parity	Nulliparous	7	0
	Primiparous	5	5
	Multiparous	0	7
Stage of gestation	Non-pregnant animals	5	1
	1st week of gestation	7	6
	>6 weeks of gestation	0	5
Age ^a^	(days)	370 ± 92	673 ± 175
Conformation ^a^	Body weight (kg)	172 ± 29	229 ± 35
	Body length (cm) ^b^	98 ± 5	110 ± 9
	Body height (cm) ^c^	87 ± 3	97 ± 5
Lameness	Mean visual score (Minimum–maximum)	0.08BBBB (0–1)	1.83BBBB (1–3)

^a^ mean ± SD; ^b^ maximal distance between the two markers on the back between shoulder blades and at the root of the tail (measured by kinematics, see Figure 2); ^c^ maximal distance between the two markers on a front foot and between shoulder blades (measured by kinematics, see Figure 2)

**Table 2:** Lattice design.

Replication I				Replication II				Replication III				Replication IV			
**A**	1	2	3	**D**	1	4	7	**G**	1	5	9	**J**	1	8	6
**B**	4	5	6	**E**	2	5	8	**H**	7	2	6	**K**	4	2	9
**C**	7	8	9	**F**	3	6	9	**I**	4	8	3	**L**	7	5	3

**A**–**F** refers to the animal (block); 1–9 refers to the treatment (9 combinations of floor design)

**Table 3:** Gait characteristics (least square means) of sound gilts and lame sows on the control floors.

Measurement	Sound Gilts		Lame Sows		SEM	*p*-Value		
	Week 1	Week 2	Week 1	Week 2		Group	Week	Interaction
**Animal**								
Walking speed, m.s^−1^	0.85	0.90	0.76	0.83	0.04	*0.075*	**0.040**	0.67
Mean back angle, °	172	172	165	167	1.6	**0.011**	0.25	0.16
**Front limbs**								
Stride length, cm	88.0	91.0	88.8	91.8	3.6	0.88	**0.018**	0.98
Foot height, cm	4.4	5.0	4.7	4.9	0.31	0.73	0.10	0.31
Swing time, ms	430	416	446	422	12	0.49	0.019	0.53
Stance time, ms	604	591	726	657	31	**0.019**	*0.079*	0.22
Swing carpal angle, °	171	166	172	169	1.8	0.30	**0.006**	0.57
Stance carpal angle, °	197	193	199	196	1.7	0.18	**0.035**	0.52
Swing carpal angle amplitude, °	58	60	52	52	3.0	0.11	0.37	0.47
Stance carpal angle amplitude, °	12.8	13.0	13.7	13.4	0.8	0.53	0.99	0.64
**Rear limbs**								
Stride length, cm	87.5	89.6	88.2	91.4	3.4	0.79	*0.055*	0.68
Foot height, cm	5.2	5.3	5.1	4.9	0.38	0.58	0.85	0.52
Swing time, ms	442	423	439	423	11	0.91	*0.088*	0.89
Stance time, ms	593	587	731	662	29	**0.008**	*0.054*	*0.098*
Swing tarsal angle, °	162	162	157	164	2.1	0.61	**0.003**	**0.010**
Stance tarsal angle, °	158	158	151	157	2.1	0.15	**0.013**	**0.010**
Swing tarsal angle amplitude, °	29	29	31	31	1.7	0.43	0.82	0.98
Stance tarsal angle amplitude, °	11.9	13.2	12.5	10.8	0.8	0.40	0.66	**0.013**

bold: significant difference (*p* < 0.05); italics: tendency (*p* < 0.10)

**Table 4:** Effects of slat and gap widths on the gait on sound gilts walking on concrete slats with parallel or perpendicular orientation.

Slat Width, mm	85			105			125			SEM	*p*-Value		
Gap Width, mm	19	22	25	19	22	25	19	22	25		Slat	Gap	Slat × Gap
**Parallel orientation**													
**Animal**													
Walking speed, m.s^-1^	0.92	0.82	0.90	0.95	0.88	0.90	0.94	0.83	0.89	0.05	0.70	0.07	0.98
**Front limbs**													
Foot height ^a^, cm	4.1	5.3	4.5	6.1	5.8	4.3	4.0	4.3	4.9	0.46	0.047	0.35	0.06
Stance time ^b^, ms	524	597	539	538	582	566	520	601	558	35	0.95	0.08	0.96
Swing carpal angle amplitude, °	58.7	58.7	56.9	60.4	59.7	53.7	55.5	54.4	61.8	3.0	0.91	0.93	0.08
Stance carpal angle amplitude, °	14.1	14.5	12.8	10.7	14.2	11.4	12.8	14.0	13.3	1.0	0.048	0.031	0.34
**Rear limbs**													
Stride length ^c^, cm	84	85	90	92	89	84	87	84	89	3.8	0.021	0.063	0.012
Foot height ^d^, cm	4.9	5.6	6.3	5.8	6.9	6.0	6.0	5.2	5.0	0.68	0.035	0.16	0.017
Stance time, ms	535	615	562	531	577	543	526	571	532	31	0.49	0.07	0.97
**Perpendicular orientation**													
**Animal**													
Mean back angle, °	171	169	170	172	171	169	173	170	170	1.3	0.58	0.040	0.44
**Front limbs**													
Stride length, cm	88.6	85.7	89.1	92.9	88.7	85.3	90.5	86.5	90.2	2.89	0.69	0.095	0.25
Foot height, cm	4.8	5.0	4.2	6.2	5.9	5.0	4.7	4.5	4.9	0.49	0.014	0.28	0.39
Swing time, ms	410	393	389	421	434	410	402	402	429	14	0.07	0.98	0.20
Swing carpal angle ^a^, °	171	175	167	166	165	169	172	171	171	3.0	0.05	0.72	0.22
Stance carpal angle ^a^, °	198	201	196	194	192	196	198	197	196	2.8	0.06	0.94	0.47
**Rear limbs**													
Stance tarsal angle amplitude, °	10.0	11.3	9.8	12.3	12.3	9.9	12.2	12.6	10.4	1.0	0.21	0.06	0.83

Bold: significant difference (*p* < 0.05); ^a^ hmax as covariate; ^b^ lmax as covariate; ^c^ hmax and lmax×treatment as covariate; ^d^ lmax and lmax × treatment as covariate.

**Table 5:** Effects of slat and gap widths on the gait on lame sows walking on concrete slats with parallel or perpendicular orientation.

Slat Width, mm	85			105			125			SEM	*p*-Value		
Gap Width, mm	19	22	25	19	22	25	19	22	25		Slat	Gap	Slat × Gap
**Parallel orientation**													
**Animal**													
Mean back angle, °	164	167	165	167	164	166	168	165	164	2.3	0.88	0.35	0.08
**Rear limbs**													
Swing time ^a^, ms	425	396	400	407	413	418	435	424	417	13	0.08	0.27	0.33
**Perpendicular orientation**													
**Front limbs**													
Foot height, cm	3.6	4.7	5.7	4.8	4.4	5.4	4.5	4.8	4.8	0.45	0.75	0.018	0.13
Swing carpal angle, °	177	174	170	170	175	171	167	174	175	2.9	0.77	0.32	0.097
Stance carpal angle, °	204	204	196	199	201	196	192	200	201	2.8	0.26	0.25	0.06
Swing carpal angle amplitude, °	53.6	54.6	55.7	51.2	45.6	50.3	57.7	60.2	50.9	4.8	0.027	0.76	0.21
**Rear limbs**													
Swing tarsal angle amplitude, °	33.2	35.1	31.7	30.8	27.6	29.2	35.1	33.4	33.6	2.5	0.015	0.62	0.68
Stance tarsal angle amplitude, °	9.7	13.1	9.9	12.7	12.2	11.8	9.7	13.1	9.8	1.3	0.18	0.022	0.30

Bold: significant difference (*p* < 0.05); ^a^ lmax as covariate.

## References

[1] National Farm Animal Care Council (2014). Code of Practice for the Care and Handling of Pigs.

[2] Anil S.S., Anil L., Deen J., Baidoo S.K., Walker R.D. (2007). Factors associated with claw lesions in gestating sows. J. Swine Health Prod..

[3] Gjein H., Larssen R.B. (1995). Housing of pregnant sows in loose and confined systems--a field study. 3. The impact of housing factors on claw lesions. Acta Vet. Scand..

[4] Heinonen M., Oravainen J., Orro T., Seppa-Lassila L., Ala-Kurikka E., Virolainen J., Tast A., Peltoniemi O.A.T. (2006). Lameness and fertility of sows and gilts in randomly selected loose-housed herds in finland. Vet. Rec..

[5] Cador C., Pol F., Hamoniaux M., Dorenlor V., Eveno E., Guyomarc’h C., Rose N. (2014). Risk factors associated with leg disorders of gestating sows in different group-housing systems: A cross-sectional study in 108 farrow-to-finish farms in france. Prev. Vet. Med..

[6] KilBride A.L., Gillman C.E., Green L.E. (2010). A cross-sectional study of prevalence and risk factors for foot lesions and abnormal posture in lactating sows on commercial farms in england. Anim. Welf..

[7] Jørgensen B. (2003). Influence of floor type and stocking density on leg weakness, osteochondrosis and claw disorders in slaughter pigs. Anim. Sci..

[8] Aarnink A.J.A., Van Den Berg A.J., Keen A., Hoeksma P., Verstegen M.W.A. (1996). Effect of slatted floor area on ammonia emission and on the excretory and lying behaviour of growing pigs. J. Agric. Eng. Res..

[9] Vermeij I., Enting J., Spoolder H.A.M. (2009). Effect of Slatted and Solid Floors and Permeability of Floors in Pig Houses on Environment, Animal Welfare and Health and Food Safety; a Review of Literature.

[10] Aarnink A.J.A., Swierstra D., Van Den Berg A.J., Speelman L. (1997). Effect of type of slatted floor and degree of fouling of solid floor on ammonia emission rates from fattening piggeries. J. Agric. Eng. Res..

[11] Ye Z., Zhang G., Seo I.H., Kai P., Saha C.K., Wang C., Li B. (2009). Airflow characteristics at the surface of manure in a storage pit affected by ventilation rate, floor slat opening, and headspace height. Biosyst. Eng..

[12] Courboulay V., Eugène A., Delarue E. (2009). Welfare assessment in 82 pig farms: Effect of animal age and floor type on behaviour and injuries in fattening pigs. Anim. Welf..

[13] Broom D.M., Gunn M., Edwards S., Wechsler B., Algers B., Spoolder H., Madec F., Von Borell E., Olsson O. (2005). The Welfare of Weaners and Rearing Pigs: Effects of Different Space Allowances and Floor Types.

[14] Jensen P., Von Borell E., Broom D.M., Csermely D., Dijkhuizen A.A., Hylkema S., Edwards S.A., Madec F., Stamataris C. (1997). The Welfare of Intensively Kept Pigs.

[15] Rähse E., Hoy S. (2007). Investigations on frequency and severity of different claw lesions in fattening pigs with regard to housing conditions. Prakt. Tierarzt.

[16] Falke A., Friedli K., Gygax L., Wechsler B., Sidler X., Weber R. (2018). Effect of rubber mats and perforation in the lying area on claw and limb lesions of fattening pigs. Animal.

[17] Council of the European Union (2008). Council Directive 2008/120/ec of 18 December 2008 on Laying Down Minimum Standards for the Protection of Pigs.

[18] Webb N.G. (1984). Compressive stresses on, and the strength of the inner and outer digits of pigs’ feet, and the implications for injury and floor design. J. Agric. Eng. Res..

[19] Young M.G., Tokach M.D., Aherne F.X., Main R.G., Dritz S.S., Goodband R.D., Nelssen J.L. (2005). Effect of sow parity and weight at service on target maternal weight and energy for gain in gestation. J. Anim. Sci..

[20] European Food Safety Authority (2005). Opinion of the scientific panel on animal health and welfare (ahaw) on a request from the commission related to welfare of weaners and rearing pigs: Effects of different space allowances and floor. Efsa J..

[21] Sasaki Y., Ushijima R., Sueyoshi M. (2015). Field study of hind limb claw lesions and claw measures in sows. Anim. Sci. J..

[22] Tubbs R.C. (1988). Lameness in sows: Solving a preventable problem. Vet. Med..

[23] Canadian Council on Animal Care (2009). Guidelines on the Care and Use of Farm Animals in Research, Teaching and Testing.

[24] Main D.C.J., Clegg J., Spatz A., Green L.E. (2000). Repeatability of a lameness scoring system for finishing pigs. Vet. Rec..

[25] Conte S., Bergeron R., Gonyou H., Brown J., Rioja-Lang F.C., Connor L., Devillers N. (2014). Measure and characterization of lameness in gestating sows using force plate, kinematic, and accelerometer methods. J. Anim. Sci..

[26] Grégoire J., Bergeron R., D’Allaire S., Meunier-Salaün M.-C., Devillers N. (2013). Assessment of lameness in sows using gait, footprints, postural behaviour and foot lesion analysis. Animal.

[27] Anil S.S., Anil L., Deen J. (2005). Evaluation of patterns of removal and associations among culling because of lameness and sow productivity traits in swine breeding herds. J. Am. Vet. Med Assoc..

[28] Stavrakakis S., Guy J.H., Syranidis I., Johnson G.R., Edwards S.A. (2015). Pre-clinical and clinical walking kinematics in female breeding pigs with lameness: A nested case-control cohort study. Vet. J..

[29] Heinonen M., Peltoniemi O., Valros A. (2013). Impact of lameness and claw lesions in sows on welfare, health and production. Livest. Sci..

[30] Mohling C.M., Johnson A.K., Coetzee J.F., Karriker L.A., Abell C.E., Millman S.T., Stalder K.J. (2014). Kinematics as objective tools to evaluate lameness phases in multiparous sows. Livest. Sci..

[31] von Wachenfelt H., Pinzke S., Nilsson C. (2009). Gait and force analysis of provoked pig gait on clean and fouled concrete surfaces. Biosyst. Eng..

[32] Thorup V.M., Tøgersen F.A., Jørgensen B., Jensen B.R. (2007). Biomechanical gait analysis of pigs walking on solid concrete floor. Animal.

[33] von Wachenfelt H., Pinzke S., Nilsson C., Olsson O., Ehlorsson C.-J. (2008). Gait analysis of unprovoked pig gait on clean and fouled concrete surfaces. Biosyst. Eng..

[34] Hoffman A.C., Moore D.A., Vanegas J., Wenz J.R. (2014). Association of abnormal hind-limb postures and back arch with gait abnormality in dairy cattle. J. Dairy Sci..

[35] Flower F.C., De Passillé A.M., Weary D.M., Sanderson D.J., Rushen J. (2007). Softer, higher-friction flooring improves gait of cows with and without sole ulcers. J. Dairy Sci..

[36] Franco-Gendron N., Bergeron R., Curilla W., Conte S., DeVries T., Vasseur E. (2016). Investigation of dairy cattle ease of movement on new methyl methacrylate resin aggregate floorings. J. Dairy Sci..

[37] Kramer J., Keegan K.G., Wilson D.A., Smith B.K., Wilson D.J. (2000). Kinematics of the hind limb in trotting horses after induced lameness of the distal intertarsal and tarsometatarsal joints and intra-articular administration of anesthetic. Am. J. Vet. Res..

[38] Buchner H.H.F., Savelberg H.H.C.M., Schamhardt H.C., Barneveld A. (1996). Limb movement adaptations in horses with experimentally induced fore- or hindlimb lameness. Equine Vet. J..

[39] Clayton H.M. (2016). Horse species symposium: Biomechanics of the exercising horse. J. Anim. Sci..

[40] Hodson E., Clayton H.M., Lanovaz J.L. (2001). The hindlimb in walking horses: 1. Kinematics and ground reaction forces. Equine Vet. J..

[41] Hodson E., Clayton H.M., Lanovaz J.L. (2000). The forelimb in walking horses: 1. Kinematics and ground reaction forces. Equine Vet. J..

[42] Thorup V.M., Laursen B., Jensen B.R. (2008). Net joint kinetics in the limbs of pigs walking on concrete floor in dry and contaminated conditions. J. Anim. Sci..

[43] Applegate A.L., Curtis S.E., Groppel J.L., McFarlane J.M., Widowski T.M. (1988). Footing and gait of pigs on different concrete surfaces. J. Anim. Sci..

[44] von Wachenfelt H., Pinzke S., Nilsson C., Olsson O., Ehlorsson C.J. (2009). Force analysis of unprovoked pig gait on clean and fouled concrete surfaces. Biosyst. Eng..

[45] Tanida H., Miura A., Tanaka T., Yoshimoto T. (1996). Behavioral responses of piglets to darkness and shadows. Appl. Anim. Behav. Sci..

[46] Grandin T. (1990). Design of loading facilities and holding pens. Appl. Anim. Behav. Sci..

